# The Role of RaxST, a Prokaryotic Sulfotransferase, and RaxABC, a Putative Type I Secretion System, in Activation of the Rice XA21-Mediated Immune Response

**DOI:** 10.1155/2014/532816

**Published:** 2014-10-19

**Authors:** Pamela C. Ronald

**Affiliations:** Department of Plant Pathology and the Genome Center, University of California, Davis, CA 95616, USA

## Abstract

Tyrosine sulfation is an important posttranslational modification that determines the outcome of serious diseases in plants and animals. We have recently demonstrated that the plant pathogen *Xanthomonas oryzae* pv. *oryzae (Xoo)* carries a functional sulfotransferase (RaxST). raxST is required for activation of rice Xa21-mediated immunity indicating the critical, but unknown, function of raxST in mediating the *Xoo*/rice interaction. The raxST gene resides in the same operon (raxSTAB) as components of a predicted type I secretion and processing system (RaxA and RaxB). These observations suggest a model where RaxST sulfates a molecule that contains a leader peptide, which is cleaved by the peptidase domain of the RaxB protein and secreted outside the bacterial cell by the RaxABC T1SS.

## 1. The Importance of Tyrosine Sulfation in Mediating Receptor-Ligand Interactions

The modification of primary and secondary metabolites by the addition or removal of sulfate can have a profound influence on their biological properties. It is estimated that about 1% of all tyrosines in eukaryotic cells are sulfated [1]. Typically, sulfated molecules are directed outside the cell, where they serve as mediators of protein-protein interactions. These interactions affect processes such as immunity and development [2]. Notable examples include sulfation of a small peptide, phytosulfokine, which is recognized by the phytosulfokine receptor to control various developmental processes in plants [3], the sulfation of Nod factors in* Sinorhizobium* species [4, 5], and sulfation of CCR5, which is the human chemokine coreceptor for CD4 [6]. Sulfation of tyrosine residues in the N-terminal segment of CCR5 appears to be critical for binding of the gp120 subunit of the envelope glycoprotein of the human immunodeficiency virus (HIV) [7]. In this review I address the critical role of sulfation in mediating the interaction of a bacterial phytopathogen with the rice XA21 pattern recognition receptor (PRR). The microbial molecule that activates XA21-mediated immunity has not yet been isolated [8].

## 2. XA21 Confers Broad-Spectrum Resistance to the Bacterial Pathogen* Xanthomonas oryzae* pv.* oryzae* (*Xoo*)

Perception of extracellular signals by cell surface receptors is of central importance to eukaryotic development and immunity [9]. Cell surface PRRs play an essential role in the innate immune responses in animals and plants [10, 11]. PRRs share conserved signaling domains, such as leucine-rich repeats (LRRs), and function via kinases, which are either integral to the receptor (plants) or associated with the receptor (animals) [12–14]. Many of these receptors regulate transcription of target genes through phosphorylation events after recognition of pathogen associated molecular patterns (PAMPs).

## 3. *Xoo* Genes Predicted to Be Involved in Small Peptide Secretion and Sulfation Are Required for Activation of XA21-Mediated Immunity (*rax *Genes)

Previous studies, using genetic approaches, led to the identification of six genes in* Xoo* falling into two functional classes, which are required for activation of XA21-mediated immunity (*rax*). Genes in the first class,* raxA*,* raxB*, and* raxC*, encode a predicted membrane fusion protein (MFP), an adenosine triphosphate (ATP) binding cassette (ABC) transporter protein, and an outer membrane protein (OMP), respectively. Together these three proteins are predicted to comprise a type I secretion system (T1SS), a structure known to be involved in secretion of molecules outside the bacterial cell [15, 16]. RaxB falls into a clade of ABC-transporters that carry a proteolytic peptidase domain in their N-termini, termed as C39, which cleaves a characteristic double glycine (GG) motif-containing signal peptide from substrates before secretion [15, 17]. These results suggest that the RaxABC T1SS is involved in the secretion of peptides/proteins and that these substrates are processed before or during secretion [18].

**Figure 1:**
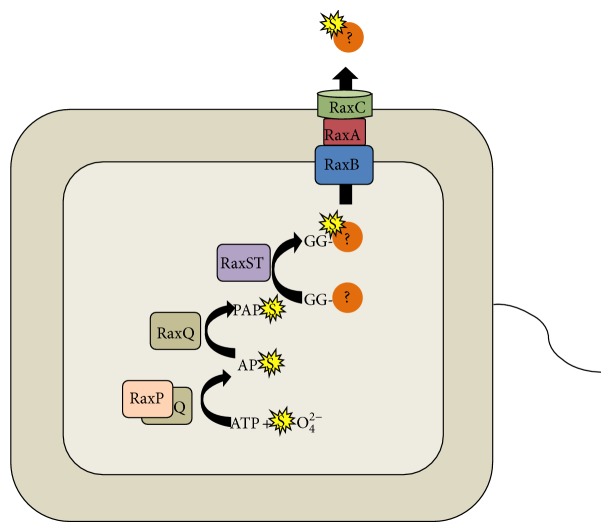
Working model for the synthesis and secretion of proteins required for activation of XA21-mediated immunity (encoded by* rax *genes). In this model, the adenosine-5′-triphosphate (ATP) sulfurylase RaxP and the adenosine-5′-phosphosulfate (APS) kinase RaxQ catalyze the production of the universal sulfuryl group donor 3′-phosphoadenosine 5′-phosphosulfate (PAPS). The RaxST sulfotransferase utilizes PAPS as a substrate. We hypothesize that RaxST sulfates a molecule that contains a leader peptide, which is cleaved by the peptidase domain of the RaxB protein and secreted outside the bacterial cell by the RaxABC T1SS.

The second class of* rax* genes is involved in sulfation.* raxP* and* raxQ, *identified through a forward genetics screen [19], encode an ATP sulfurylase and an adenosine-5′-phosphosulfate (APS) kinase. These proteins function in concert to produce 3′-phosphoadenosine 5′-phosphosulfate (PAPS), the universal sulfuryl group donor [19]. This class also includes the* raxST*-encoded sulfotransferase.* raxST* resides in the same operon with* raxA* and* raxB* (*raxSTAB*), suggesting that this group of proteins may target similar substrates and/or control a similar biological process [15]. Strains carrying mutations in these* rax *genes compromise the ability of* Xoo *to activate XA21-mediated immunity. In support of a role for these genes in a shared biological process, we and others have demonstrated that* raxST* and other* rax* genes are transcriptionally regulated by cell density, where high cell density induces and low cell density represses gene expression [20]. A model for* rax* gene function is shown in Figure 1.

## 4. *Xoo *RaxST Is a Functional Tyrosine Sulfotransferase

It has recently been shown, using a novel ultraviolet photodissociation mass spectrometry (UVPD-MS) approach, that RaxST is capable of* in vitro* tyrosine sulfotransferase activity [21]. This result indicates that tyrosine sulfation is not restricted to eukaryotic species as previously hypothesized.

## 5. Concluding Remarks

The discovery of the RaxSTAB operon and regulators of* raxSTAB* expression is an important first step in dissecting its role in mediating the interaction of* Xoo* with the host. Amino acid sequence identity examination of the* raxSTAB* genomic region in publicly available sequenced genomes reveals that orthologs are present in the agronomically important pathogens* X. oryzae* pv.* oryzicola* (*Xoc*) (98% identity), * X. campestris* pv.* vesicatoria* (*Xcv*) (88% identity),* X. axonopodis* pv.* citrumelo* (*Xac*) (88% identity), and* X. campestris* pv.* musacearum* (*Xcm*) (>85% identity). Investigations of the role of RaxSTAB in these other organisms will advance our understanding of this highly conserved but understudied operon in these important pathogens and of the role of tyrosine sulfation, a process not previously studied in bacteria, in mediating interactions with agronomically important plant hosts.

A major unknown is the identity of RaxST substrates and the function of such modified molecules. It is also unknown if this sulfation system plays a role in the interaction of bacterial pathogens with animal hosts. Furthermore, the proposed peptidase function of the RaxB protein has not been demonstrated nor has the RaxB substrate been identified.
